# Regulation of neutrophil function by the extracellular matrix

**DOI:** 10.1042/BST20253020

**Published:** 2025-10-16

**Authors:** Christopher J. Calo, Margaret Radke, Tanvi Patil, Laurel E. Hind

**Affiliations:** Department of Chemical and Biological Engineering, University of Colorado Boulder, Boulder, CO, U.S.A.

**Keywords:** antimicrobial function, extracellular matrix, extravasation, migration, neutrophil

## Abstract

Neutrophils play a critical role in maintaining healthy tissue by acting as the first cellular responders to inflammatory challenges. Unfortunately, when this response is dysregulated, defects in neutrophil function can contribute to the pathogenesis of several diseases and conditions, including cancer, fibrosis, and aberrant wound healing. Understanding the factors that regulate the neutrophil response is critical for improving disease outcomes. It is becoming increasingly appreciated that the extracellular matrix (ECM) serves as a significant regulator of the neutrophil response. The ECM is a complex network of fibrous proteins and proteoglycans that provides both physical and biochemical cues that can modulate cell behavior. Importantly, the composition, structure, and mechanics of the ECM often undergo significant changes in disease. Studies have shown that matrix stiffness and composition can alter neutrophil behavior, but our understanding of how the various structural and mechanical properties of the ECM govern the neutrophil response remains incomplete. In part, this is due to the challenges involved in isolating distinct properties of the matrix to determine their individual roles in regulating the neutrophil response. In this review, we summarize the recent efforts that have been made to better understand how ECM properties affect the neutrophil inflammatory response and offer suggestions for future directions for the field.

## Introduction

Neutrophils, the most abundant immune cell type, act as the body’s first line of cellular defense and play a crucial role in clearing infections, healing wounds, and repairing damaged tissues [1–3]. Unfortunately, dysregulated neutrophil function contributes to the pathogenesis of diseases and conditions, including cancer, fibrosis, aberrant wound healing, and aging [4–10]. The role of dysregulated neutrophil function in disease is a fascinating and complicated topic and has been reviewed in detail [11–15]. Many studies have highlighted the potential of neutrophils as therapeutic targets [16–20]. However, to effectively design such therapeutics, it is imperative to understand the regulatory factors that govern the neutrophil response. Diseases and conditions worsened by neutrophil dysregulation are often associated with significant changes in the extracellular matrix (ECM) [21–24]. Changes, such as protein deposition, crosslinking, and turnover of fibrillar matrix proteins, are dynamic and alter the mechanical and structural properties of the ECM [25–33]. Neutrophils are mechanosensitive [34,35], but our understanding of how physical changes to the ECM affect their function remains incomplete.

The ECM is a complex network present in all tissues primarily comprises two macromolecules: fibrous proteins and proteoglycans [36]. Together, they provide scaffolding for cells along with biochemical and biomechanical cues crucial for a diverse range of cellular functions, including differentiation, homeostasis, proliferation, adhesion, and migration [37,38]. The composition of the ECM and the roles of the individual components have been extensively reviewed elsewhere and will not be a focus of this review [39–41]. In brief, fibrous proteins consist mainly of collagens, elastin, fibronectin, and laminin, which provide structural integrity to the ECM and facilitate cell adhesion and motility [40,41]. As mechanosensing cells with receptors that bind these proteins, changes in the protein structure of the ECM have the potential to affect neutrophil functions in the ECM. Proteoglycans exist in three main categories: small leucine-rich proteoglycans, modular proteoglycans, and cell-surface proteoglycans that contribute to upstream signaling of pathways involving receptor tyrosine kinases, the insulin-like growth factor-I receptor, and Toll-like receptors [39]. Neutrophils express these receptors on their surfaces [42–45], suggesting proteoglycans could alter neutrophil function.

In this review, we will discuss recent efforts to identify how the ECM modulates the neutrophil inflammatory response and discuss future directions for further understanding how the ECM regulates neutrophil behavior.

## Extravasation

**Figure 1:**
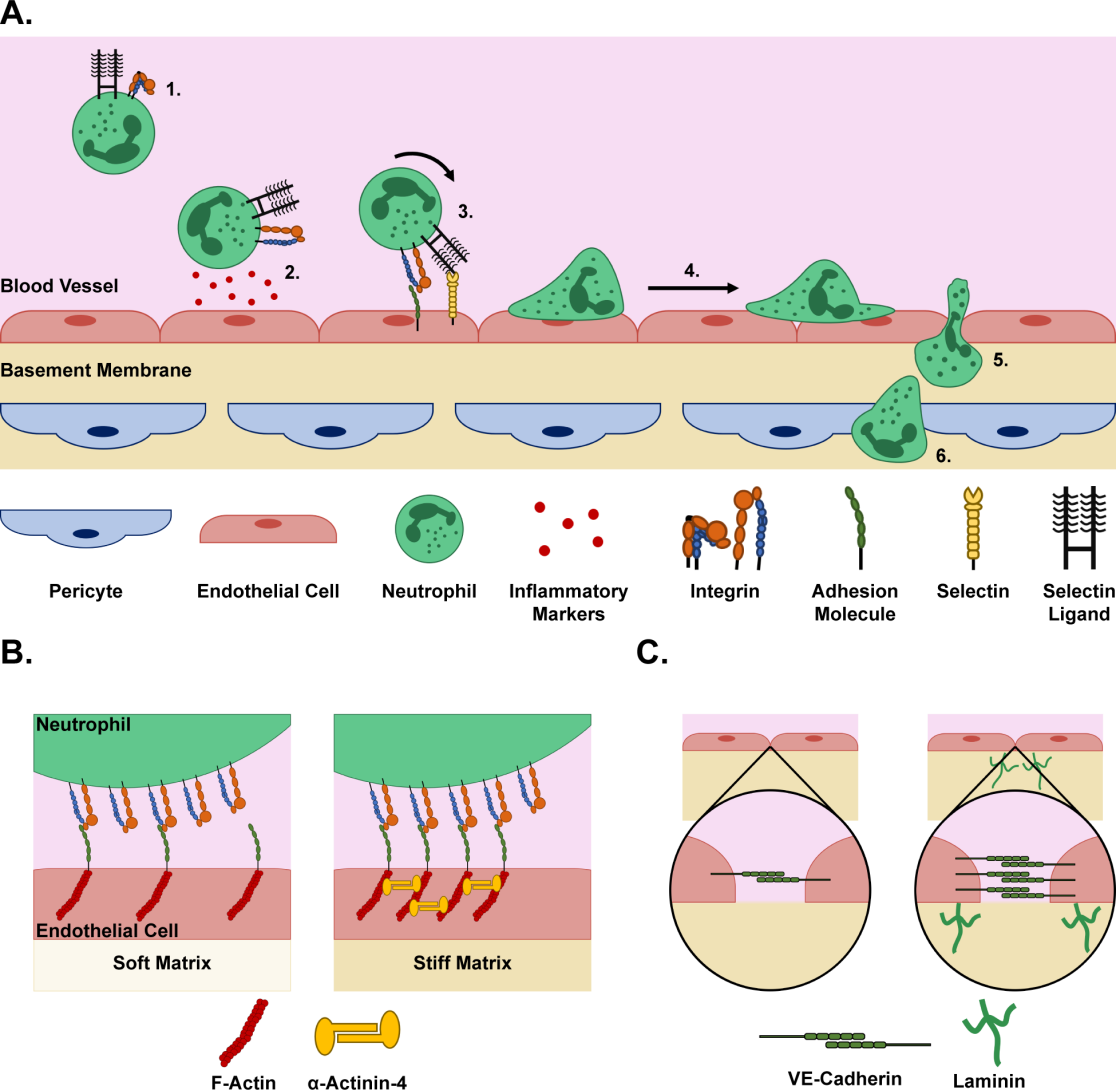
The neutrophil extravasation process. **A**. (1) Neutrophils circulate in the blood in an inactive state (2). Upon an inflammatory challenge in the surrounding tissue, endothelial cells that line the lumens of blood vessels secrete inflammatory markers that recruit and activate neutrophils (3). Activated neutrophils begin to bind to the endothelium via integrin-adhesion molecule and selectin–selectin ligand interactions as they roll along the endothelium in the direction of blood flow, accumulating binding interactions (4). Once there are enough binding interactions, the neutrophils arrest and flatten as they begin crawling along the endothelium (5). Neutrophils probe the endothelium and the underlying matrix to find a path of least resistance to extravasate out of the blood vessel (6). Finally, the neutrophils complete the extravasation process by migrating through the pericyte layer of the basement membrane to reach the inflamed tissue. **B**. Illustrative summary of findings from [46]. Endothelial cells seeded on stiff matrices exhibit increased intercellular adhesion molecule 1 (ICAM-1) clustering on the cell periphery due to increased α-actinin-4-induced filamentous actin (F-actin) clustering within the endothelial cytosol. This facilitates increased neutrophil adhesion and ultimately extravasation. **C**. Illustration of findings from [47]. The presence of laminin 511 in the basement membranes increases junctional vascular endothelial cadherin (VE-cadherin) between endothelial cells, which impairs neutrophil extravasation.

Neutrophils circulate through the blood in an inactive state. Upon an inflammatory challenge, neutrophils become activated via the leukocyte adhesion cascade. During this cascade, neutrophils tether to, roll along, adhere to, and crawl on the vascular endothelium, then migrate through the vessel wall (Figure 1A). Endothelial cells are crucial for recruiting and activating neutrophils for this process [48]. Importantly, endothelial cells are also mechanosensitive, altering their behavior in response to physical signals from the underlying ECM on which they are adhered [49–53]. Thus, the ECM can indirectly regulate the early steps of neutrophil inflammation through the endothelium.

Neutrophil tethering and rolling occur when inflamed endothelial cells express P- and E-selectins on their luminal surface and bind selectin ligands, such as P-selectin glycoprotein 1 and E-selectin ligand 1, on the neutrophil membrane [54,55]. Interestingly, MacKay and Hammer demonstrated that increasing stiffness of E-selectin-coated hydrogels resulted in enhanced monocytic cell attachment and slowed cell rolling speeds [56], but the same trend was not seen for P-selectin. As endothelial cells modulate their stiffness in response to the stiffness of their underlying substrate [57], it is possible that this same trend may hold true for neutrophil tethering *in vivo*.

During the rolling phase, chemokines bind chemokine receptors on the surfaces of neutrophils, leading to ‘inside-out’ activation of integrins, causing a conformational change that enhances their binding affinity [54,55]. This increase in integrin activity causes the transition from rolling to firm adhesion and crawling of neutrophils on the endothelium. These phases are predominantly driven by β_2_-integrins on the neutrophil surface binding to intercellular adhesion molecules (ICAMs) 1 and 2 [49,50] on the endothelial surface [54,55]. The actin-binding protein, α-actinin-4, is up-regulated in endothelial cells seeded on stiff matrices, which facilitates ICAM-1 clustering on the endothelial surface, resulting in increased neutrophil adhesion (Figure 1B) [46].

As neutrophils crawl along the endothelium, they probe the surface with lamellipodia in search of a suitable place to undergo transendothelial migration (TEM) [54]. The up-regulation of α-actinin-4 increased neutrophil TEM [46]. Specifically, α-actinin-4 facilitated a stiffness gradient across the endothelial cells, with the greatest stiffness at the cell periphery. TEM can occur via a paracellular (between two cells) or transcellular (through one cell) route; the increased stiffness at the periphery of the endothelial cells allowed for increased paracellular TEM. In addition to stiffness, studies have investigated other ECM features that regulate neutrophil TEM. Our lab recently found collagen I concentration affects the extent of neutrophil TEM in response to infection; however, whether this was solely due to a change in the endothelial cells or the pore structure of the ECM is unclear [58].

Once neutrophils pass through the endothelial lumen, they enter the basement membrane, a laminin-rich matrix that surrounds blood vessels, consisting primarily of laminin 511 and laminin 411 [47]. Recent isoform knockout studies have shown laminin preferentially regulates neutrophil TEM through endothelium barrier function. Specifically, Laminin Alpha 4 (LAMA4), the gene encoding laminin 411, knock-out mice show greater neutrophil adhesion and lower transmigration compared with Laminin Alpha 5 (LAMA5) knocked-out mice, suggesting high laminin 511 content inhibits neutrophil TEM. It is hypothesized that laminin 511 enhances VE-cadherin localization to cell–cell borders, strengthening venule barrier function, which ultimately decreases neutrophil TEM (Figure 1C) [47].

The basement membrane is largely formed by pericytes, which surround blood vessel capillaries, and through co-ordinated signaling with endothelial cells ensure vessel integrity. The ECM plays a role in how these cells interact, which can lead to changes in the neutrophil response. Endothelial cells increase secretion of macrophage migration inhibitory factor when seeded on stiff substrates [59], which decreases pericyte contractility. This results in increased transcellular holes in the pericyte layer, allowing increased neutrophil transmigration [60]. Therefore, matrix stiffness regulates neutrophil TEM through the endothelium alone and endothelial cell interactions with pericytes. Pericytes also regulate neutrophil extravasation through ECM remodeling. Prolonged stimulation of pericytes with the inflammatory markers tumor necrosis factor alpha (TNF-α) and interleukin 17A (IL-17A) results in increased collagen IV and decreased laminin deposition, facilitating enhanced TEM [61]. As noted previously, some isoforms of laminin restrict TEM, possibly due to its recruiting VE-cadherin to endothelial borders to reinforce tight junctions [47]. Thus, it is possible that the prolonged exposure of pericytes to TNF-α and IL-17A, resulting in decreased laminin production in the basement membrane, allows for greater neutrophil TEM via endothelial tight junction weakening. Together, these studies demonstrate the capability of the ECM-endothelial cell-pericyte axis as a regulator of neutrophil inflammation.

The ECM regulates neutrophil activation and extravasation largely through phenotypic alterations of the endothelium. Several studies have investigated the impact of ECM stiffness on the endothelium and, subsequently, on the neutrophil response, but there are still unanswered questions on what aspects of the leukocyte adhesion cascade are altered by stiffness. Outside of stiffness, the effects of other matrix properties, such as pore structure and protein composition, are not completely understood. The effects from pore structure should be isolated from the often coupled changes in stiffness or protein concentration. Some efforts have investigated the effects of laminin isoforms on TEM, but this research should be validated by activating neutrophils with cytokines that do not up-regulate neutrophil adhesion, such as IL-1β and IL-6, which are known to increase neutrophil TEM and migration [60]. Additionally, future studies are needed to fully characterize laminin’s role in TEM and the mechanism by which it alters endothelium barrier function.

## Migration

Following extravasation, neutrophils must quickly navigate highly diverse ECMs throughout the body to reach the site of inflammation. To do so, they follow chemical gradients of danger signals produced near the inflammatory site. Neutrophils interact extensively with the local ECM during the migration process: probing the pores to find the path of least resistance, degrading dense portions of matrix, and binding matrix proteins [62,63]. Matrix stiffness dictates the force exerted back on neutrophils when they interact with the matrix, the size and shape of the pores that neutrophils move through govern how cells migrate, and viscoelasticity determines how deformable the pores are to neutrophils [64,65].

Neutrophils achieve a rapid response by using amoeboid migration [66], which does not rely on proteolytic degradation and has low adhesion to the ECM [67]. Neutrophils become polarized when transmigrating the endothelium, with lamellipodia at the leading edge of the cell and a uropod in the rear [64,68]. Neutrophils migrate by contracting the uropod [64,68]. The magnitude of the forces from the uropod is affected by substrate stiffness, which also impacts migration speed, cell spreading, and intracellular signaling [62,64]. In recent years, it has been found that neutrophils migrate using the nucleus as a mechanical gauge to probe different paths before committing to the path of least resistance, and this migration is protease-independent [63,69].

Matrix composition is known to alter neutrophil migration, but it is difficult to parse out how different matrix properties affect migration. Initial research in the field focused on how different ECM components affect neutrophil behavior [69–71]. Neutrophils possess several integrin receptors that can bind different types of ECM proteins, which could lead to differential signaling pathways being activated inside the neutrophils [72,73]. When comparing collagen I, collagen III, fibrin, and agarose, neutrophil migration distance and displacement were inhibited in collagen III [70]. Collagen III-rich tissue is a common outcome in patients with acute respiratory distress syndrome (ARDS), a disease with significantly dysregulated neutrophil function [74,75]. In fact, the presence of procollagen III, the precursor of collagen III, in patients with persistent ARDS is associated with poor outcomes [75]. This diminished neutrophil migration and an acceleration of effector functions, discussed below, by collagen III may prevent neutrophils from migrating far enough away from blood vessels before performing their effector functions, contributing to the vascular leakage of blood into air sacs in ARDS. When comparing collagen I to a mixture of decellularized basement membrane combined with collagen I, fewer cells migrated into the collagen I matrix than the mixed matrix, and the neutrophils migrated a shorter distance [69]. This is likely due to a variety of changes between the matrices, including different proteins, matrix mechanics, pore size, and number of cell binding sites. Matrix stiffness has previously been shown to alter neutrophil migration on two-dimensional (2D) surfaces; there is a biphasic relationship between stiffness and migration speed, with neutrophils migrating fastest at intermediate stiffnesses [76].

**Figure 2:**
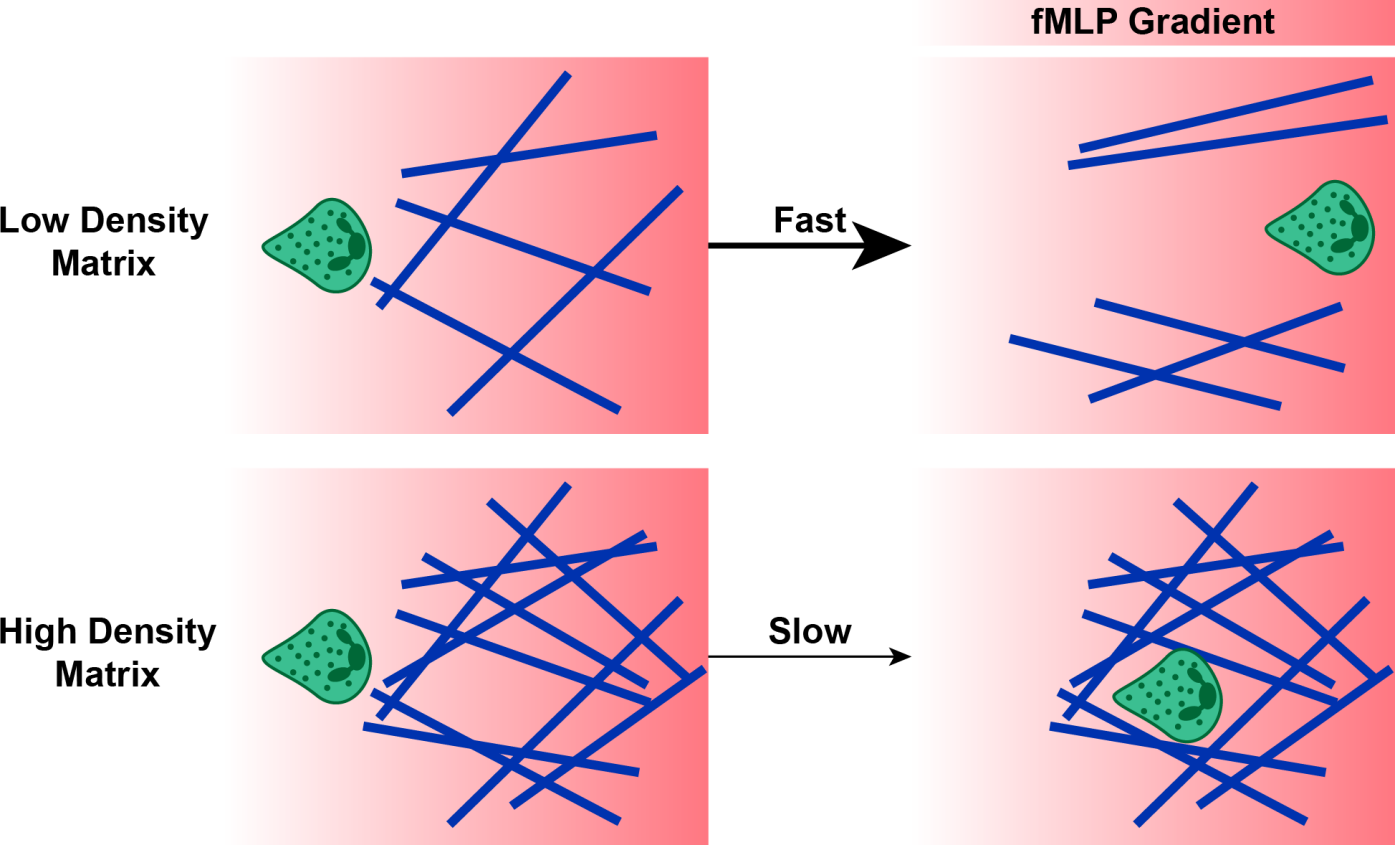
Schematic illustration summarizing findings from [78] . In low-protein-density ECMs, neutrophils deform the matrix to allow for faster and more linear migration along a chemotactic gradient of fMLP, compared with neutrophils in protein-dense ECMs.

Pore size determines whether neutrophils can migrate through a material, and the largest part of a neutrophil that must fit through a pore is the nucleus. When pores have cross sections smaller than 1–2 μm^2^, the nucleus cannot deform to fit through the pore, and cells cannot migrate [77]. In pores with areas of 3–4 μm^2^, neutrophils can migrate with the assistance of proteolytic enzymes. One way to alter pore size *in vitro* is by changing the concentration of fibrous proteins in the matrix. In collagen I matrices, lower protein concentrations have larger pores, and neutrophils migrate further [58] and faster [58,78]. Furthermore, lower matrix concentrations lead to increased neutrophil deformation of the ECM, allowing for more linear migration along the chemotactic gradient (Figure 2) [78]. In higher concentrations, fewer pores are large enough for the neutrophil nucleus to fit through, and the cells have more difficulty physically deforming the matrix as they push through the pores, so neutrophils turn more while migrating, leading to a lower chemotactic index [78].

While altering matrix protein concentration is biologically relevant, doing so changes several matrix properties at once, including pore size, stiffness, and binding site availability. Future work should aim to develop systems that decouple these properties, allowing for investigations of how the isolated properties regulate neutrophil migration, and these studies should be in three dimensions (3Ds) since many of the initial studies investigating how stiffness impacts neutrophils were done on 2D hydrogels. Additionally, viscoelasticity, or the deformability of a matrix, should be investigated since it is altered in many diseases previously mentioned, such as cancer and fibrosis, also characterized by neutrophil dysfunction [79,80]. Human tissue and native ECM components are viscoelastic; however, the effects of viscoelasticity on cell behavior are still not well understood [81]. Since neutrophils migrate by physically deforming their matrix, viscoelasticity is likely to influence neutrophil migration. Additionally, research into ECM molecules only focuses on how different ECM proteins affect neutrophil migration, with no research on the role proteoglycans—which have previously been shown to directly affect cell proliferation [82]—play in neutrophil migration. To better understand neutrophil dysregulation, especially in diseases also characterized by ECM dysregulation, future research should investigate how the tissue environment that neutrophils migrate through can alter neutrophil behavior and contribute to neutrophil dysregulation.

## Effector functions

Upon arrival at the inflammatory site, neutrophils perform effector functions, including phagocytosing pathogens and necrotic cell debris, producing reactive oxygen species (ROS), and generating neutrophil extracellular traps (NETs). These processes are critical for clearing pathogens but also have the potential to cause damage to surrounding host cells and, therefore, must be tightly regulated. Biochemical and mechanical signals from the ECM are one avenue through which these functions are regulated.

Neutrophils use phagocytosis to clear pathogens and cell debris from an inflammatory site. Phagosomes form when neutrophils engulf a particle, then granules within the neutrophil fuse with the phagosome to deliver antimicrobial molecules. Simultaneously, NADPH oxidase complexes form on the phagosome’s membrane to induce ROS production [83]. Additionally, the pH in the phagosome acidifies briefly before a prolonged alkalinization [84]. These processes are susceptible to changes in the pH of the ECM, which is altered in cancer and chronic wounds [85–88]. For example, neutrophils increase endocytosis in acidic environments (pH 6.0) compared with neutral environments (pH 7.4) but exhibit decreased bactericidal capabilities [89]. The mechanism by which this occurs is not known but may be the result of less ROS production in the acidic environment.

Inflammatory cytokines, such as TNF-α and IL-1β, affect phagocytic activity of neutrophils [90]. Interestingly, increasing the stiffness of neutrophils’ underlying substrate increases their secretion of these cytokines [91]. Additionally, functionalizing the substrates with fibronectin and, to a lesser extent, collagen and laminin further increased cytokine secretion by neutrophils on the stiffer substrates [91]. This suggests ECM mechanics and composition can regulate neutrophil phagocytosis through altered cytokine secretion.

Neutrophils perform NETosis by decondensing their DNA and releasing it in a web-like structure containing bactericidal factors to ensnare pathogens to prevent the spread of infections. However, NETosis induces a pro-inflammatory response implicated in the pathogenesis of cancer, COVID-19, rheumatoid arthritis, and sepsis [92]. Therefore, efforts have been made to understand the regulatory factors that govern NETosis to treat these diseases through NET inhibition. Diseases associated with NETosis involve mechanical and compositional changes to the ECM. To investigate how matrix stiffness and cell adhesion affect NETosis, Erpenbeck et al. seeded neutrophils on polyacrylamide gels of varying Young’s moduli [93]. They found that as the stiffness of the underlying substrate increased, so did phosphatidylinositol 3-kinase (PI3K)-dependent lipopolysaccharide (LPS)-induced NETosis. Blocking neutrophil adhesion significantly decreased NETosis in response to LPS, demonstrating the importance of cell adhesion for the process. Similarly, neutrophils seeded on polydimethylsiloxane (PDMS) substrates of increasing stiffness had increased NET formation [91], and coating the PDMS surface with collagen, fibronectin, laminin, or vitronectin increased NET formation on the stiffer substrates, with fibronectin coating causing the largest increase. NETosis was subsequently impaired by inhibiting focal adhesion kinase (FAK). Furthermore, neutrophils seeded on ECMs with up-regulated collagen I, as seen in cirrhotic ECMs, induced NETs, which prevented T cells from attacking hepatocellular carcinoma [94]. Together, these studies show that stiffer ECMs, often seen in diseased tissue, lead to increased NETosis in an adhesion-dependent manner.

Neutrophils are among the first cells at the site of inflammation; one of their roles is to secrete inflammatory cytokines and chemokines to recruit other immune cells. These inflammatory cytokines, including TNF-α and IL-1β, are coupled with NET formation [91,95], and the cytokines affect phagocytic activity of neutrophils [90]. Abaricia et al. investigated how substrate stiffness and protein coatings alter neutrophil secretions of the pro-inflammatory cytokines and chemokines TNF-α, IL-1β, C-C motif chemokine ligand 2 (CCL2), CCL3, CCL5, and C-X-C motif chemokine Ligand 1 (CXCL1) [91]. They found that increasing the stiffness of neutrophils’ underlying substrate increases their secretion of these cytokines and the formation of NETs, which aligns with previously discussed research showing that NETosis increases on stiffer substrates [93]. Additionally, functionalizing the substrates with fibronectin and, to a lesser extent, collagen and laminin further increased cytokine secretion by neutrophils on the stiffer substrates [91]. The same authors previously showed that neutrophils on wetted surfaces decrease the production of these same cytokines compared with smooth or rough surfaces [95], but the effect of substrate properties on neutrophil secretion is understudied.

At the site of inflammation, neutrophils perform a respiratory burst, triggering the formation of NADPH oxidase complexes to generate ROS in both phagosomes and outside the cell. Subsequent degranulation leads to the release of enzymes, such as myeloperoxidase (MPO), that also produce ROS [83]. ROS also serve regulatory roles through oxidation of kinases, phosphatases, and proteases, affecting signaling pathways for neutrophil migration, antimicrobial functions, and cytokine release [96–98]. The regulatory capabilities of ECM composition and stiffness on ROS production are therefore of interest. Increasing the stiffness of neutrophils’ underlying matrix led to increased release of MPO by the neutrophils [91]. MPO release increased even more when stiffer substrates were coated with fibronectin. These differences were found to be FAK signaling dependent. Using Ibidi μ-slide chemotaxis chambers, Kraus et al. investigated the inflammatory response of neutrophils encapsulated in either collagen I, collagen III, agarose, or fibrin gels to a source of N-formyl-methionyl-leucyl-phenylalanine (fMLP) [70]. Interestingly, neutrophils reached peak ROS production earlier in collagen III gels than the others, but the overall ROS production was not increased. As previously mentioned, different ECM proteins bind with different integrin receptors on neutrophils, which can lead to different neutrophil responses [72,73]. It is possible that binding collagen III activated the protein kinase C (PKC) signaling pathway to form the NADPH oxidase complex or mitochondrial activation, which could explain the earlier production of ROS [99]. These studies clearly demonstrate that ECM composition and mechanics influence neutrophil ROS production; however, the exact mechanisms through which this is achieved are still not fully understood.

**Figure 3:**
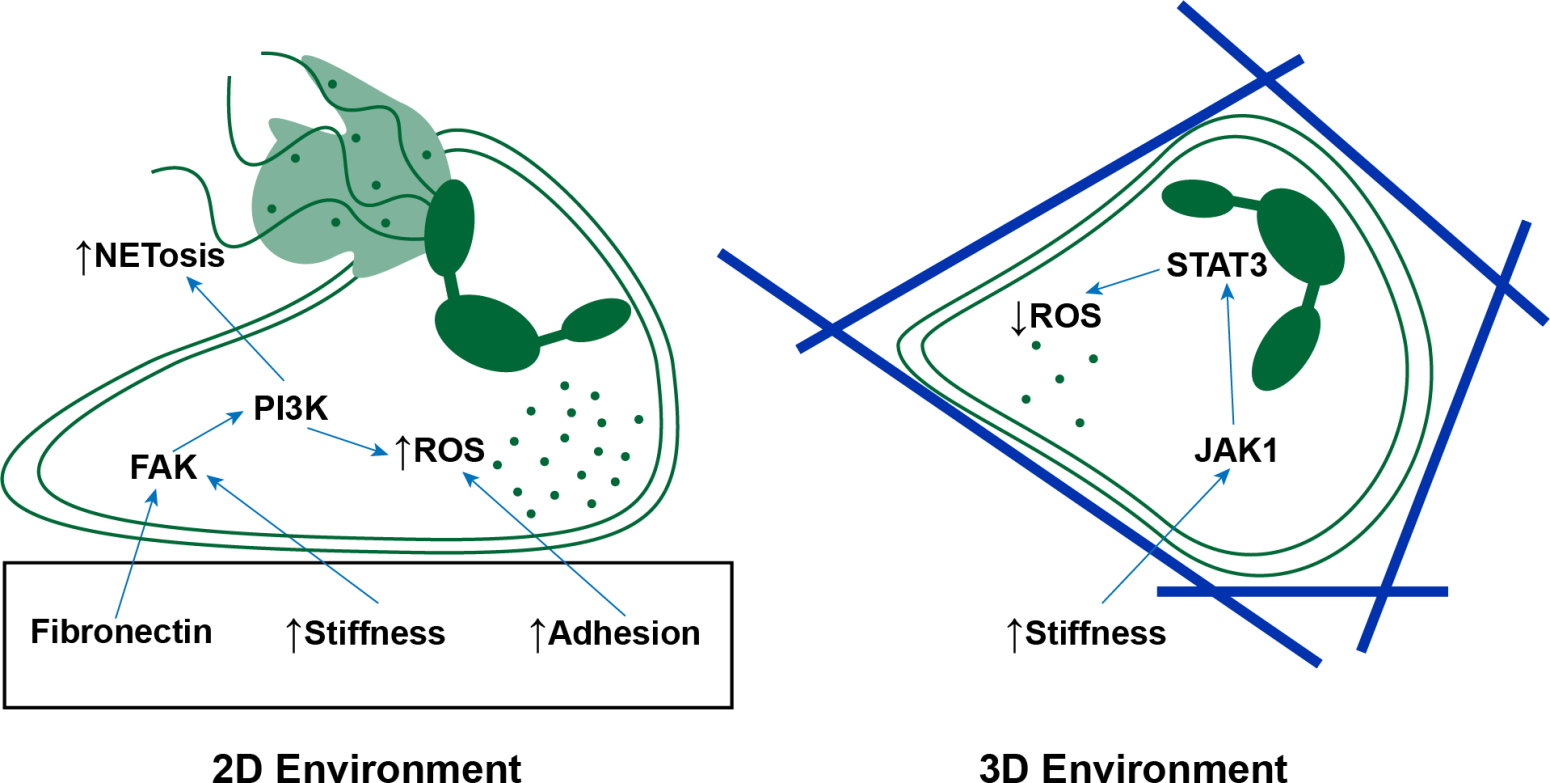
Schematic illustration summarizing findings from [91] and [101] r. Neutrophils in 2D environments increase reactive oxygen species (ROS) production and neutrophil extracellular trap formation (NETosis) when the surface stiffness increases and when the surface is coated with fibronectin through the focal adhesion kinase/phosphatidylinositol 3-kinase (FAK/PI3K) signaling pathway. Stronger neutrophil adhesion to the 2D surface also increases ROS production. In 3D environments, neutrophils decrease ROS production as the surrounding matrix stiffness increases in a Janus kinase 1/Signal transducer and activator of transcription 3 (JAK1/STAT3) signaling-dependent manner.

Due to the nonspecific nature of functions like NETosis, ROS production, and ECM degradation, neutrophils are traditionally known as inflammatory cells that cause collateral damage to the surrounding tissue. However, in recent years, emerging evidence shows that they also contribute to inflammatory resolution and tissue repair [100]. As with the pro-inflammatory functions, these anti-inflammatory processes are also modulated by the ECM. By encapsulating neutrophils in gelatin methacryloyl of varying stiffnesses, Jiang et al. found that neutrophils seeded in higher stiffness gels adopted a more anti-inflammatory phenotype compared with those in the softer gels [101]. Conditioned media from neutrophils in the stiffest gels facilitated faster wound closure by human umbilical vein endothelial cells (HUVECs) in a scratch assay and promoted more endothelial sprouting than conditioned media from the softer gels. These metrics suggest the potential for the neutrophils to promote vascularization. In contrast with Abaricia et al. they found that neutrophils in the higher stiffness gels produced less ROS [91]. This may be the result of encapsulating the neutrophils in a 3D system versus seeding them on a 2D system. This could lead to different distributions of cell surface receptors and ultimately affect the mechanosensing of the cells [102]. Interestingly, Jiang et al. found the changes in neutrophil phenotype due to changes in stiffness relied on the Janus kinase 1/signal transducer and activator of transcription 3 (JAK1/STAT3) signaling pathway, as opposed to the focal adhesion kinase (FAK)/PI3K signaling pathway. The difference in signaling pathways identified may give credence to the idea that the difference in neutrophil adhesion in the 2D and 3D systems may result in differential mechanosensing of the ECM resulting in different responses by the neutrophils (Figure 3).

Neutrophils perform effector functions to clear pathogens and necrotic tissue. Recent studies have shown these processes are, in part, regulated by several aspects of the ECM, such as pH, protein composition, stiffness, and matrix dimensionality. These efforts have identified the FAK/PI3K and JAK1/STAT3 signaling pathways as potential therapeutic targets for diseases worsened by dysregulated neutrophil functions. The studies have shown how fibrous matrix proteins affect ROS production and NETosis. However, there should be further investigation into the signaling pathways that allow for ECM regulation of ROS production, which could produce a novel therapeutic target for diseases with aberrant ROS production. Additionally, the other major macromolecules in the ECM, proteoglycans, are poorly studied. Binding of Toll-like receptor 4 (TLR4) on the neutrophil surface by pathogen-associated molecular patterns, such as LPS, is known to induce ROS and NETosis [103,104]. Furthermore, heparan sulfate proteoglycans have been reported to activate inflammatory responses in mouse microglia through TLR4 signaling [105]. How these and other proteoglycans regulate antimicrobial functions of neutrophils is an interesting question that merits further investigation.

## Conclusion

The ECM is a highly diverse and dynamic network that provides chemical and physical signals to modulate a host of cellular processes, including homeostasis, proliferation, migration, differentiation, cell adhesion, and tissue remodeling. Recent efforts have begun to elucidate specific mechanisms through which the ECM regulates neutrophil extravasation, migration, and effector functions during an inflammatory response. Historically, much of this research has been performed using 2D systems, so these results should be validated in physiologically relevant 3D systems that include relevant cell types, such as endothelial cells when studying neutrophil extravasation. Additionally, much of this previous research was achieved with synthetic hydrogels, such as polyacrylamide, that lack relevant matrix protein signaling and create pores too small for subsequent neutrophil migration. Conversely, natural polymers, such as collagen I, are soft and do not mimic the physiological stiffness of *in vivo* tissue matrices. Future studies should utilize materials that can replicate relevant stiffnesses while also incorporating relevant matrix proteins and physiological pore size to further study the neutrophil response.

While many studies use natural polymers, especially in 3D research, isolating the impact of stiffness, pore size, and protein concentration can be difficult. For example, stiffness is often modulated through polymer concentration, which will also affect pore size and the number of binding sites. To best understand how each of these properties might alter neutrophil behavior, a material with tunable properties and pores large enough to support neutrophil migration should be used. This may be achieved by interpenetrating hydrogel networks where one network is composed of natural matrix proteins and the other is a synthetic material that can be used to tailor the overall network mechanics.

Lastly, there are few studies on the effects of proteoglycans or viscoelasticity on the neutrophil response. These are important components and properties of the ECM that are under-researched. Studies have shown that proteoglycans regulate neutrophil effector functions, and viscoelasticity has been shown to influence cell migration and behavior. These are both potential avenues through which the ECM may regulate neutrophil functions that should be thoroughly investigated.

Recently, it has been appreciated that neutrophils not only play an important role in protecting the body from pathogens and assisting in wound healing but also contribute to the pathology of many diseases. Therefore, researchers are trying to develop therapeutics that target neutrophils. However, this requires a better understanding of the factors that contribute to the neutrophil response and result in the neutrophil dysregulation seen in disease. These diseases are often accompanied by an abnormal ECM, and the ECM has been shown to have a profound impact on cell behavior. Further research understanding how ECM properties affect neutrophils may lead to a better understanding of both a healthy neutrophil response and the dysregulation response seen in these diseases.

Regulation of neutrophil function by the ECM is still a highly understudied topic, and the future benefit of studying these interactions is immense. Understanding how both the biochemical and physical properties of the matrix contribute to the neutrophil response will allow us to better understand the pathology of a range of diseases and conditions, opening the potential for the development of new therapeutics. Furthermore, the tools developed to investigate these interactions, from new biomaterials to new imaging modalities, will benefit the fields of bioengineering, tissue engineering, immunology, and beyond.

**Perspectives**

- How neutrophils interact with their surrounding microenvironment may be leveraged to treat diseases caused or worsened by neutrophil dysfunction.
- The extracellular matrix (ECM) is a highly diverse and dynamic environment. The structure and mechanics of the ECM provide regulatory signals during the neutrophil inflammatory response, but our understanding of the extent to which this occurs, and the mechanisms employed, remains incomplete.
- Development of systems that allow for isolated control over key ECM properties, such as pore size, protein density, elasticity, etc., could lead to the identification of novel therapeutic targets to treat neutrophil dysfunction.

## Abbreviations

ARDS: acute respiratory distress syndrome
CCL: C-C motif chemokine ligand
CXCL: C-X-C motif chemokine ligand
2D: two dimension
3D: three dimension
ECM: extracellular matrix
FAK: focal adhesion kinase
F-actin: filamentous actin
HUVEC: human umbilical vein endothelial cell
ICAMs: intercellular adhesion molecules
IL: interleukin
JAK1: Janus kinase 1
LAMA4: Laminin Alpha 4
LAMA5: Laminin Alpha 5
LPS: lipopolysaccharide
MPO: myeloperoxidase
NETs: neutrophil extracellular traps
PDMS: polydimethylsiloxane
PI3K: phosphatidylinositol 3-kinase
PKC: protein kinase C
ROS: reactive oxygen species
STAT3: signal transducer and activator of transcription 3
TEM: transendothelial migration
TLR4: Toll-like receptor 4
TNF-α: tumor necrosis factor alpha
VE-cadherin: vascular endothelial cadherin
fMLP: N-formyl-methionyl-leucyl-phenylalanine
